# Supplementary material for: Characterisation of the Viral Community Associated with the Alfalfa Weevil (Hypera postica) and Its Host Plant, Alfalfa (Medicago sativa)
Source: Viruses. 2021 Apr 28;13(5):791. doi: 10.3390/v13050791 (PMC8145008; doi:10.3390/v13050791)
Supplement: Supplementary file 1 [file viruses-13-00791-s001.zip › viruses-1172408-supplementary.pdf]

Supplementary figure 1: Rarefaction curves of viral communities at the family level.

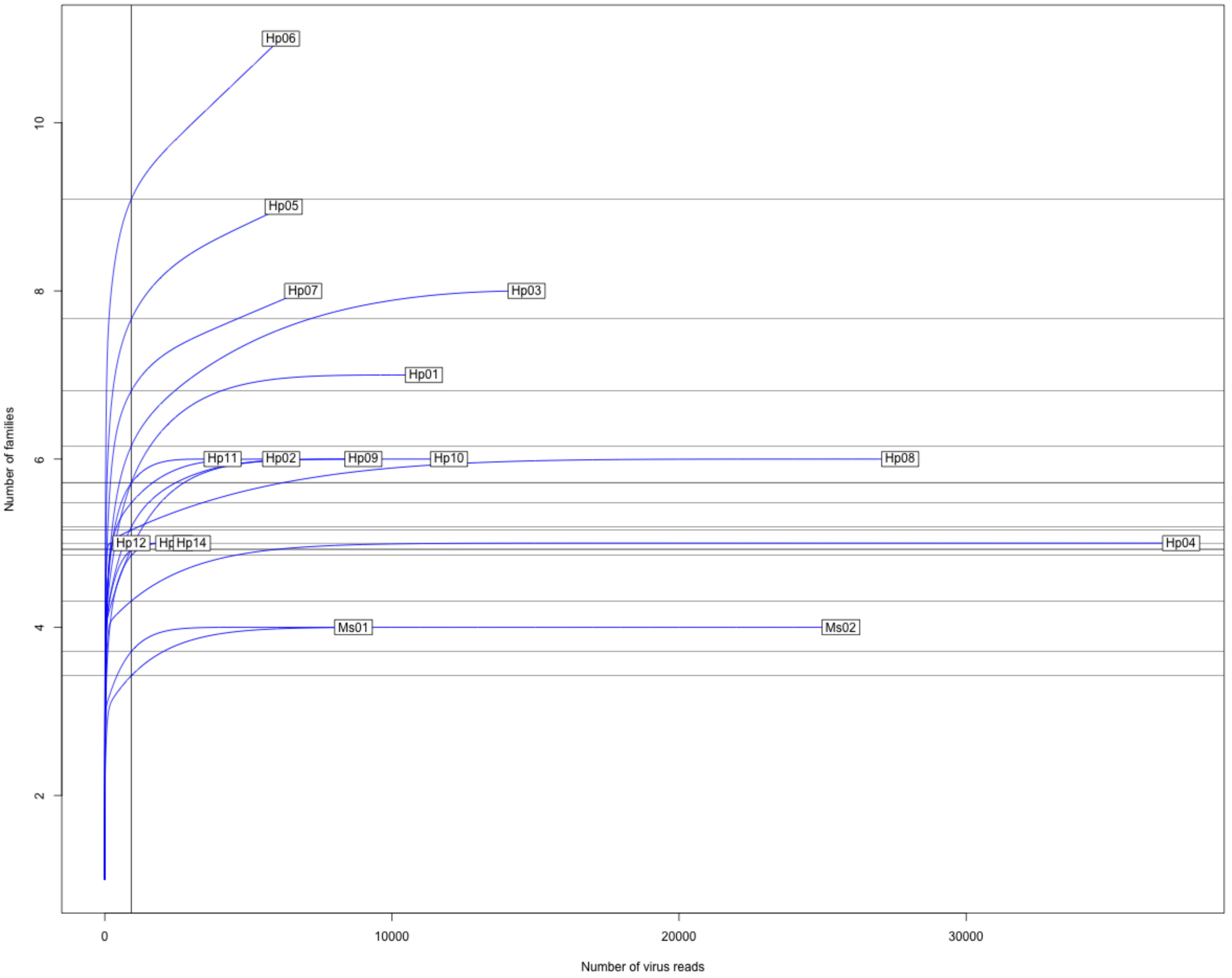

Supplementary figure 2: Proportion of reads classified into arthropod-infecting families (red) and plant or fungus-infecting families (blue). Hp: Hypera postica samples, Ms: Medicago sativa samples.

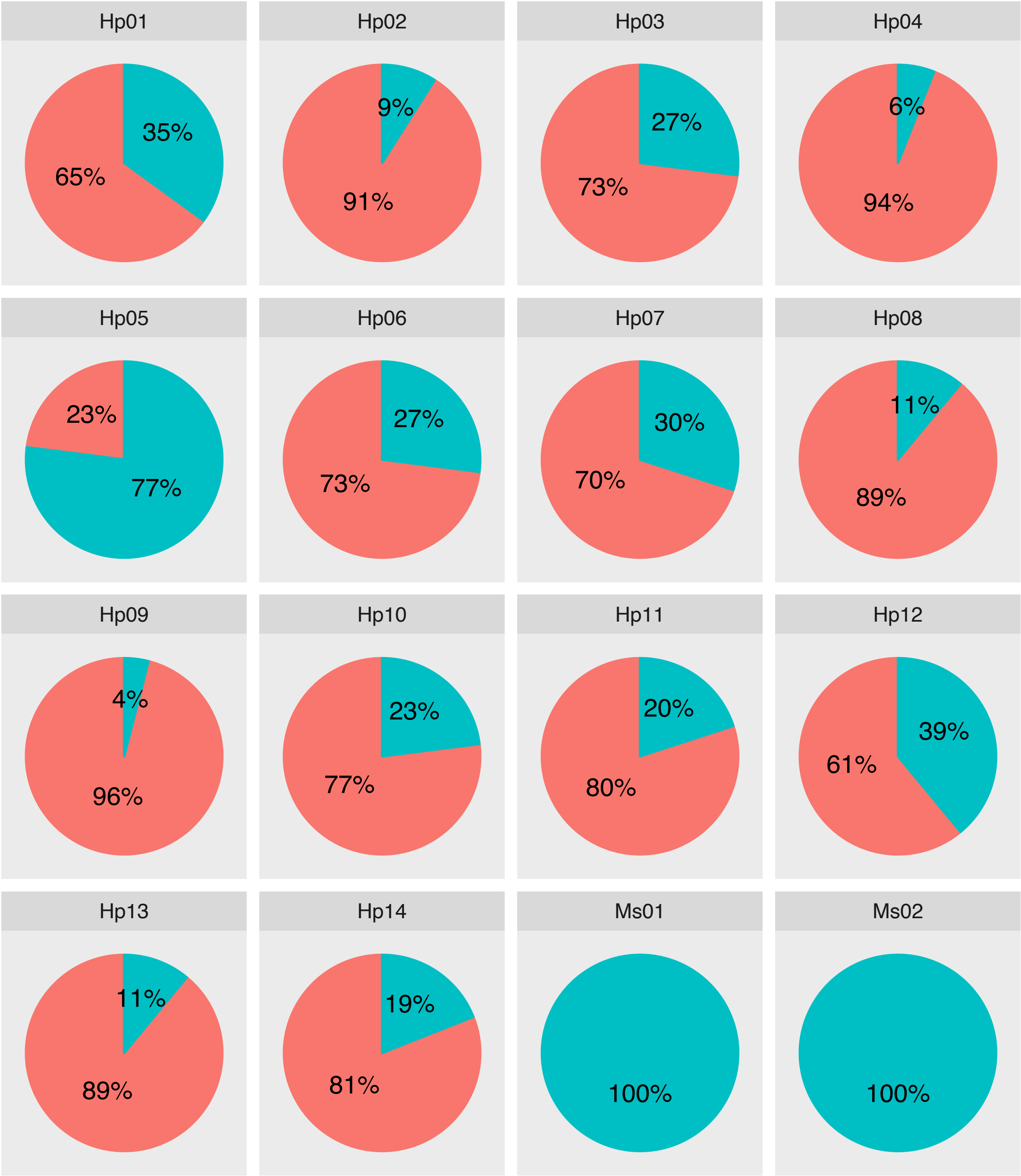

Supplementary figure 3: Maximum likelihood phylogenetic tree based on (a) the first segment and on (b) the third segment of Alfalfa mosaic virus genomes, including the one isolated during this study (in red).
The tree was mid-point rooted. Bootstrap values (1000 replicates) superior to 50% are indicated at each node. Scale bar corresponds to nucleotide substitutions per site.

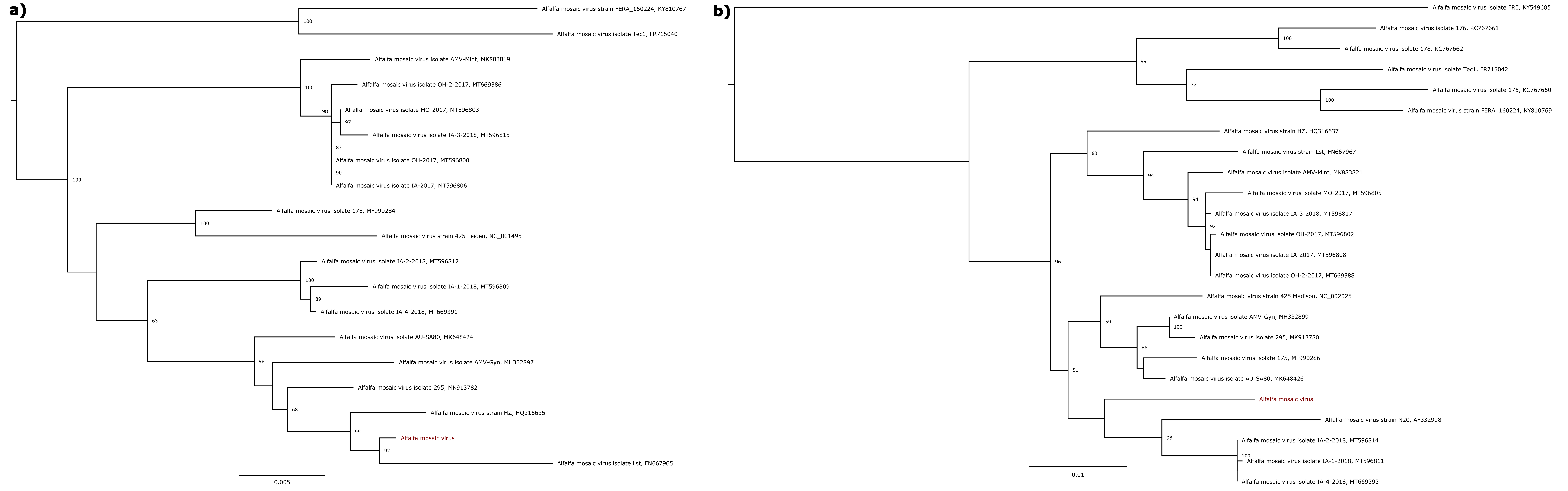

Supplementary figure 4: Maximum likelihood phylogenetic tree based on (a) the RNA-dependent RNA polymerase protein and on (b) the capsid protein of 31 amalgaviruses, in-cluding the one isolated during this study (in red). The tree was mid-point rooted. Bootstrap val-ues (1000 replicates) superior to 50% are indicated at each node. Scale bar corresponds to amino acid substitutions per site.

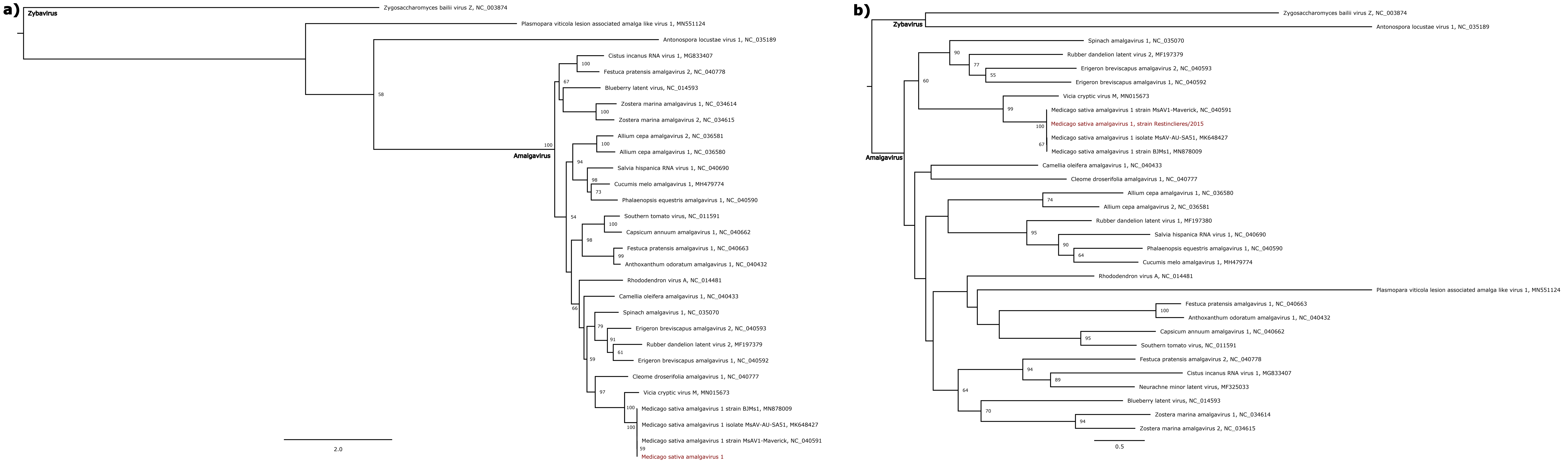

Supplementary figure 5: Maximum likelihood phylogenetic tree based on (a) the first segment and on (b) the second segment of alfalfa infecting partitiviruses, including the ones isolated dur-ing this study (in red).
The tree was mid-point rooted. Bootstrap values (1000 replicates) superior to 50% are indicated at each node. Scale bar corresponds to nucleotide substitutions per site.

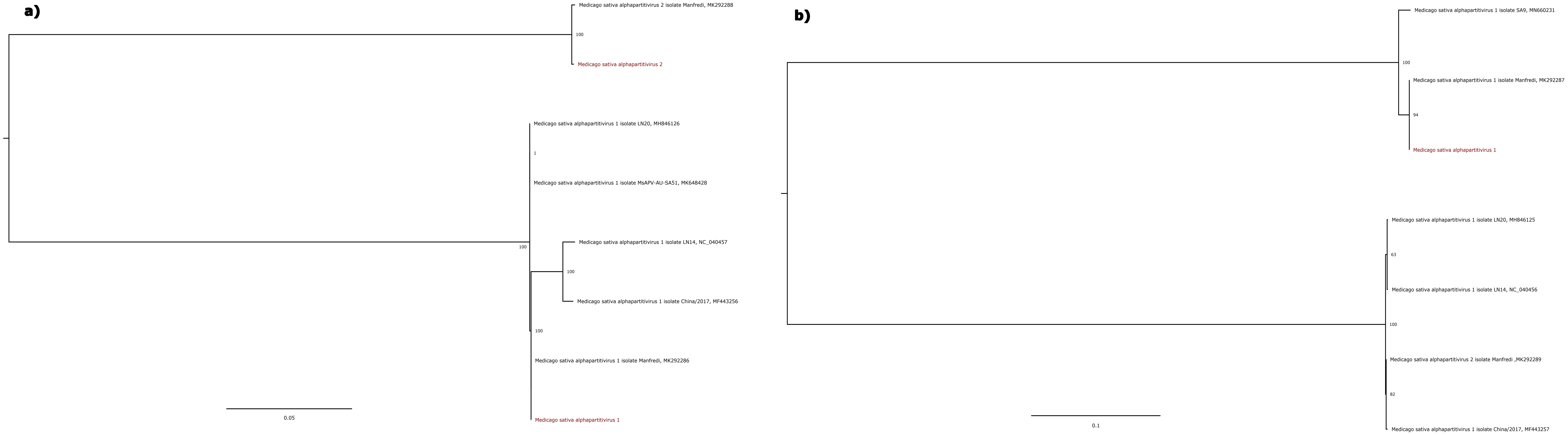

Supplementary figure 6: Maximum likelihood phylogenetic tree based on the whole genome of 6 Bean leafroll virus isolates, including the one isolated during this study (in red). The tree was mid-point rooted. Bootstrap values (1000 replicates) superior to 50% are indicated at each node. Scale bar corresponds to nucleotide substitutions per site.

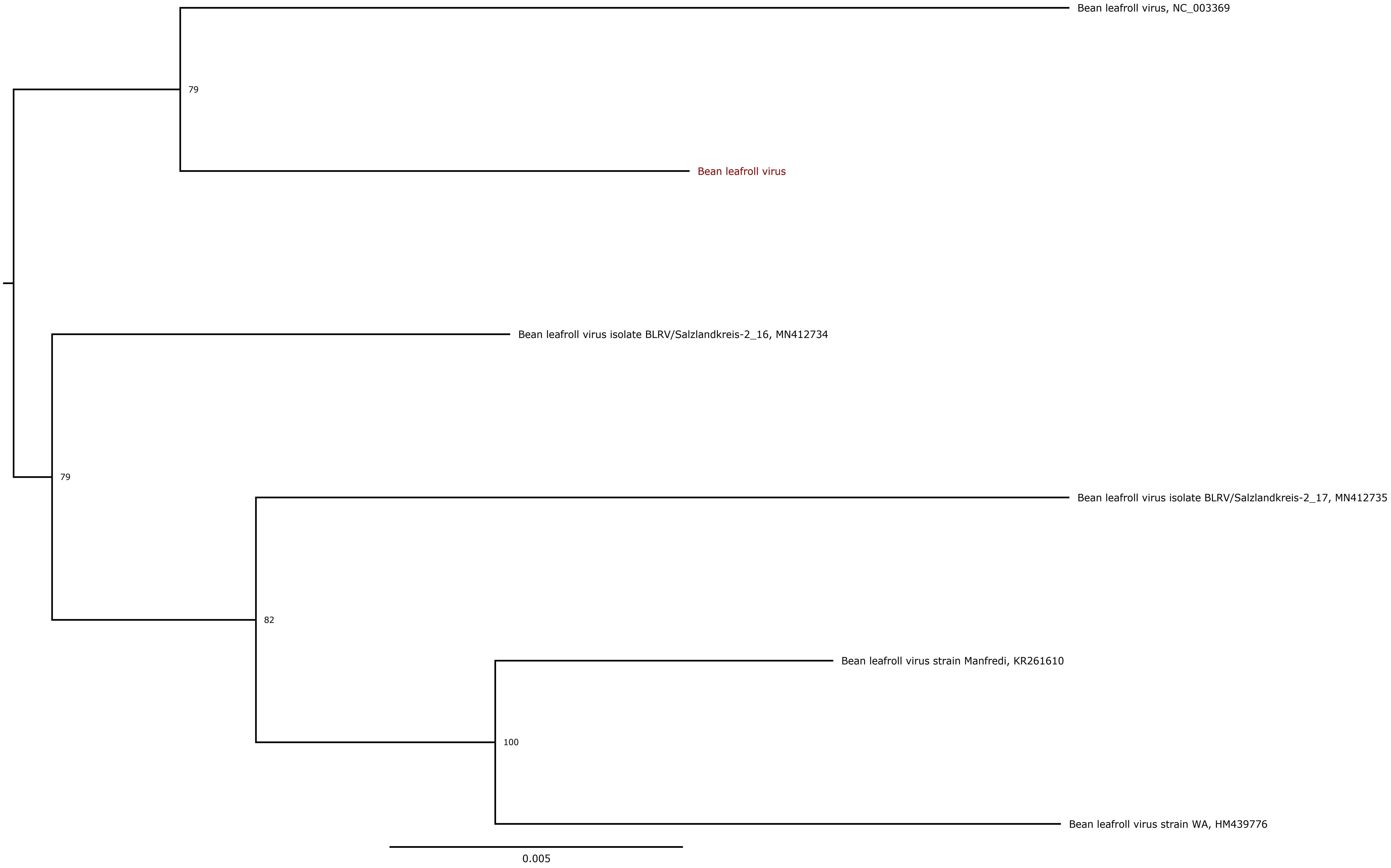

Supplementary figure 7: Maximum likelihood phylogenetic tree based on (a) the RNA-dependent RNA polymerase protein and on (b) the capsid protein of 38 sinaiviruses. The virus reported in this study is marked in red. The tree was mid-point rooted. Bootstrap values (1000 replicates) superior to 50% are indicated at each node. Scale bar corresponds to amino acid substitutions per site.

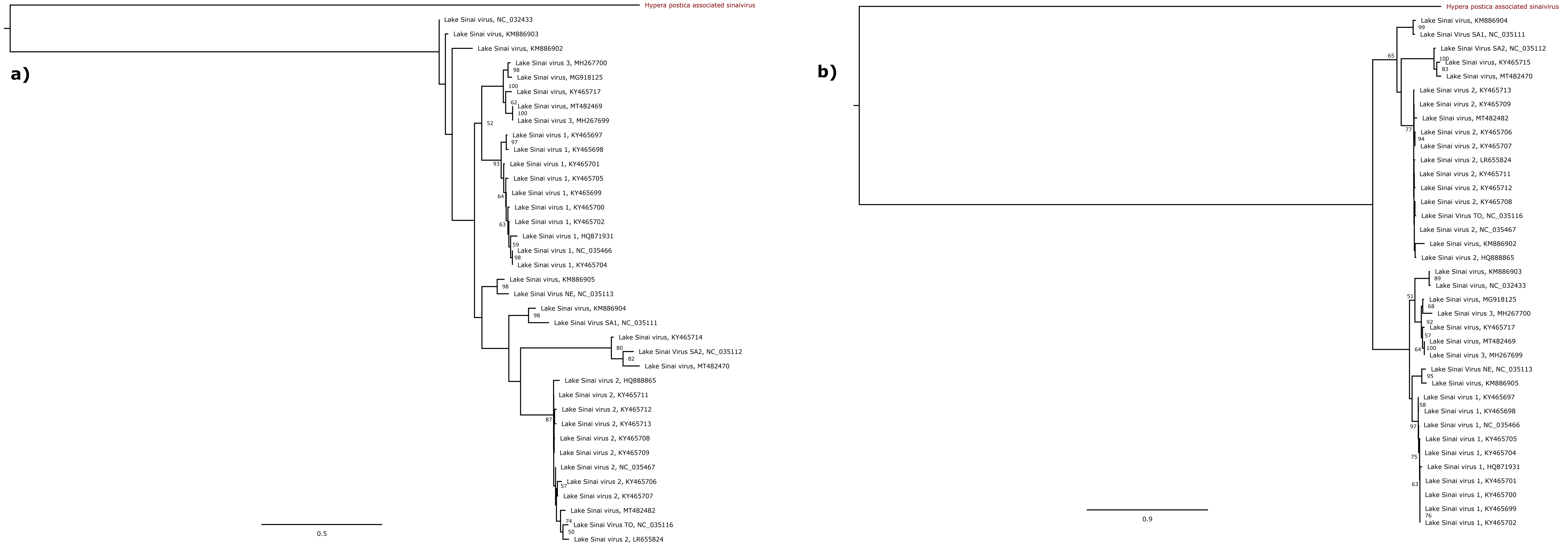

Supplementary figure 8: Maximum likelihood phylogenetic tree based on the polyprotein of 9 unclassified RNA viruses. The virus reported in this study is marked in red. The tree was mid-point rooted. Bootstrap values (1000 replicates) superior to 50% are indicated at each node. Scale bar corresponds to amino acid substitutions per site.

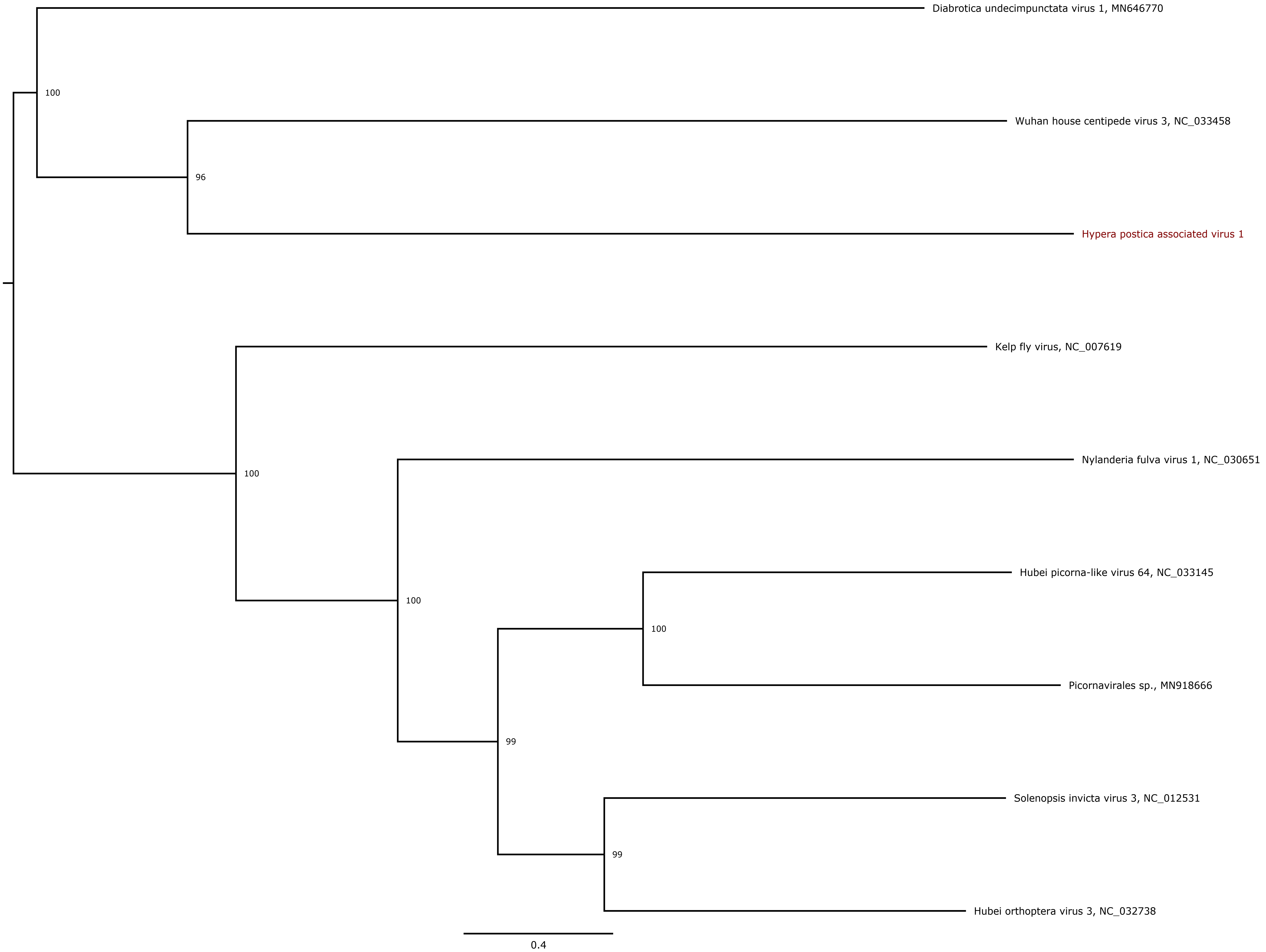

Supplementary table 1: Collected samples and their associated metadata.

sample	species	agroecosystem	date	weight (mg)	number of cleaned reads
Hp01	Hypera postica	Crop	01/04/2015	1251	146785
Hp02	Hypera postica	Crop	01/04/2015	973	97378
Hp03	Hypera postica	Crop	01/04/2015	1194	154278
Hp04	Hypera postica	Crop	01/04/2015	1222	495681
Hp05	Hypera postica	Crop	01/04/2015	1115	109493
Hp06	Hypera postica	Crop	01/04/2015	1076	96267
Hp07	Hypera postica	Crop	01/04/2015	1583	130843
Hp08	Hypera postica	Crop	01/04/2015	1897	337236
Hp09	Hypera postica	Crop	01/04/2015	250	205297
Hp10	Hypera postica	Crop	01/04/2015	500	314646
Hp11	Hypera postica	Crop	01/04/2015	750	109247
Hp12	Hypera postica	Meadow	01/04/2015	1328	124123
Hp13	Hypera postica	Meadow	01/04/2015	1223	122774
Hp14	Hypera postica	Meadow	01/04/2015	1144	263429
Ms01	Medicago sativa	Crop	01/04/2015	1000	84257
Ms02	Medicago sativa	Meadow	01/04/2015	1000	269822

Supplementary table 2: Viral reads counts contingency tables (family scale). The reads were classified to the family level using DIAMOND taxonomic assignment, as described in the material and methods section.

Taxonomy	Hp01	Hp02	Hp03	Hp04	Hp05	Hp06	Hp07	Hp08	Hp09	Hp10	Hp11	Hp12	Hp13	Hp14	Ms01	Ms02
Alphaflexiviridae	8	44	70	1129	21	0	66	455	0	1059	397	26	0	84	0	0
Amalgaviridae	458	0	302	0	765	236	0	0	0	0	0	0	0	0	1405	17351
Birnaviridae	0	0	0	0	0	0	0	0	0	0	0	0	4	0	0	0
Bromoviridae	30	4	26	15	61	26	8	5	4	6	5	0	0	0	11	0
Caulimoviridae	0	0	0	0	0	10	0	0	0	0	0	0	0	0	0	0
Endornaviridae	4	0	0	0	0	0	0	0	0	0	0	0	0	0	0	0
Geminiviridae	0	305	0	0	0	346	0	0	0	0	0	0	0	0	0	0
Iflaviridae	7795	5643	11218	35570	1680	4372	5005	25534	8086	9521	3336	603	2279	2660	0	0
Luteoviridae	0	0	0	0	253	123	428	1380	0	0	0	0	0	0	0	0
Mesoniviridae	0	0	0	0	0	1	0	0	0	0	0	0	0	0	0	0
Microviridae	0	0	0	0	0	0	0	0	0	0	0	0	0	0	0	15
Myoviridae	0	0	0	0	1	0	0	0	0	0	0	0	0	0	0	0
Partitiviridae	2817	146	3067	772	3443	771	1364	326	266	1391	344	245	117	280	6480	7760
Parvoviridae	0	0	0	0	0	247	0	0	631	0	0	0	0	0	0	0
Permutotetraviridae	0	0	0	0	0	0	36	0	0	0	0	0	0	7	0	0
Phenuiviridae	0	0	4	0	0	0	0	0	0	10	0	0	0	0	0	0
Qinviridae	0	0	0	0	5	0	0	0	0	0	0	0	0	0	0	0
Reoviridae	0	0	2	0	0	1	1	0	0	0	0	0	0	0	0	0
Secoviridae	0	0	0	0	0	0	0	0	0	0	0	0	18	0	0	0
Sinhaliviridae	0	0	0	0	0	0	0	0	0	0	0	52	30	0	0	0
Siphoviridae	0	0	0	0	0	0	0	0	17	0	0	0	0	0	0	0
Solemoviridae	302	0	6	65	88	5	0	504	0	0	0	11	76	144	0	0
Tymoviridae	0	0	0	0	0	0	0	0	0	0	20	0	0	0	0	512

Supplementary table 3: Viral reads counts contingency tables (species scale). The reads were classified to the family level using mapping (BOWTIE2), as described in the material and methods section.

Taxonomy	Hp01	Hp02	Hp03	Hp04	Hp05	Hp06	Hp07	Hp08	Hp09	Hp10	Hp11	Hp12	Hp13	Hp14	Ms01	Ms02
Alfalfa mosaic virus	76	8	51	44	121	50	22	10	8	13	12	0	0	0	23	7
Alfalfa virus F	0	0	0	0	0	0	0	0	0	0	36	0	0	43	0	927
Bean leafroll virus	38	0	195	370	376	156	631	1669	137	213	38	49	44	132	113	0
Medicago sativa alphapartitivirus 1	0	6	21	0	0	0	0	46	0	0	0	63	0	47	14	7143
Medicago sativa alphapartitivirus 2	2520	106	2489	422	2037	579	862	236	197	775	305	75	0	114	839	161
Medicago sativa amalgavirus 1	411	0	272	0	667	211	0	0	0	0	0	0	0	0	1330	15583
Hypera postica associated alphaflexivirus	36	326	471	6426	103	0	338	1812	0	9102	2274	129	0	504	0	0
Hypera postica associated sobemovirus 1	0	0	0	65	32	0	0	504	0	0	0	11	1	3	0	0
Hypera postica associated sobemovirus 2	0	0	0	0	0	5	0	0	0	0	0	0	75	141	0	0
Hypera postica associated sobemovirus 3	302	0	6	0	56	0	0	0	0	0	0	0	0	0	0	0
Hypera postica associated iflavirus 1	18808	13758	26443	82541	3942	10408	11738	63195	19275	22418	7638	1408	5412	6404	0	0
Hypera postica associated iflavirus 2	9	5	442	3686	24	131	254	118	0	645	96	0	117	15	0	0
Hypera postica associated permutotetravirus	0	3	0	0	0	3	722	0	0	0	21	0	0	939	0	0
Hypera postica associated sinaivirus	0	0	0	0	0	0	0	0	0	0	0	1137	30	0	0	0
Hypera postica associated virus 1	0	0	0	0	7851	6922	7804	0	0	23678	9669	15172	14601	48108	0	0

Supplementary table 4: List of viruses reported in this study, associated information, and GenBank accession numbers.

Family	Genus	Species	Segment number (if applicable)	Complete CDS	Contig length (b)	Number of mapped reads against contig	Mean coverage	New species (ICTV demarcation criteria)	Closest relative	Percentage of Identity to closest relative	Putative host range	Accession number
Bromoviridae	Alfamovirus	Alfalfa mosaic virus	segment 1	yes	3729	4226	219	no	Alfalfa mosaic virus isolate 295	segment 1 (MK913782): 99.1% nucleotide identity	plants	MW676127
			segment 3	yes	2038	613	56	no	Alfalfa mosaic virus strain 425 Madison	segment 3 (K02703): 100.0% nucleotide identity		MW676128
Tymoviridae	Marafivirus	Alfalfa virus F	-	yes	6778	12077	445	no	Alfalfa virus F isolate SM-1_C50	whole genome (NC_040565): 100.0% nucleotide identity	plants	NC_040565
Partitiviridae	Alphapartitivirus	Medicago sativa alphapartitivirus 1	segment 1	yes	1942	4492	363	no	Medicago sativa alphapartitivirus 1 isolate Manfredi	whole segment (MK292286): 100.0% nucleotide identity	plants	MW676139
			segment 2	yes	1712	27105	3703	no		whole segment (MK292287): 100.0% nucleotide identity		MW676140
Partitiviridae	Alphapartitivirus	Medicago sativa alphapartitivirus 2	segment 1	yes	1882	130876	17170	no	Medicago sativa alphapartitivirus 2 isolate Manfredi	whole segment (MK292288): 99.8% nucleotide identity	plants	MW676141
Amalgaviridae	Amalgavirus	Medicago sativa amalgavirus 1	-	yes	3417	83418	4655	no	Medicago sativa amalgavirus 1 isolate MsAV-AU-SA51	whole genome (MK648427): 99.6% nucleotide identity	plants	MW676142
Luteoviridae	Luteovirus	Bean leafroll virus	-	yes	5965	18730	1052	no	Bean leafroll virus isolate BLRV/Salzlandkreis-2_16	whole genome (MN412734): 98.2% nucleotide identity	plants	MW676129
Alphaflexiviridae	Platypuvirus	Hypera postica associated alphaflexivirus	-	yes	7633	87469	2439	yes	Insect-associated alphaflexivirus 1	RNA-dependent RNA polymerase protein (QHB15107): 57.19% amino acid identity	plants, fungi	MW676130
Solemoviridae	Sobemovirus	Hypera postica associated sobemovirus 1	-	yes	3241	2386	201	na	Hubei sobemo-like virus 23	RNA-dependent RNA polymerase protein (YP_009330124): 41.71% amino acid identity	plants	MW676135
Solemoviridae	Sobemovirus	Hypera postica associated sobemovirus 2	-	yes	2842	976	102	na	Soybean thrips sobemo-like virus 3	RNA-dependent RNA polymerase protein (QPZ88397): 51.89% amino acid identity	plants	MW676136
Solemoviridae	Sobemovirus	Hypera postica associated sobemovirus 3	-	no	2696	2938	435	na	Hubei sobemo-like virus 29	RNA-dependent RNA polymerase protein (YP_009330084): 49.20% amino acid identity	plants	MW676137
Permutotetraviridae	na	Hypera postica associated permutotetravirus	-	yes	4788	6715	432	na	Smithfield permutotetra-like virus	RNA-dependent RNA polymerase protein (QIJ25871): 40.24% amino acid identity	arthropods	MW676133
Sinhaliviridae	Sinaivirus	Hypera postica associated sinaivirus	-	yes	5826	4620	166	na	Lake Sinai virus	RNA-dependent RNA polymerase protein (AJR19138): 39.89% amino acid identity	arthropods	MW676134
Iflaviridae	Iflavirus	Hypera postica associated iflavirus 1	-	yes	10035	1202836	28625	yes	Aulacophora lewisii iflavirus 1	polyprotein (QOW95919): 47.92% amino acid identity	arthropods	MW676131
Iflaviridae	Iflavirus	Hypera postica associated iflavirus 2	-	yes	8820	22097	1203	yes	Aulacophora lewisii iflavirus 1	polyprotein (QOW95919): 47.14% amino acid identity	arthropods	MW676132
na	na	Hypera postica associated virus 1	-	yes	12255	822063	14597	yes	Wuhan house centipede virus 3	polyprotein (YP_009342325): 36.55% amino acid identity	arthropods	MW676138
